# The Cuspid Tooth in Its Relation to Character

**Published:** 1899-06

**Authors:** Frank L. Schattuck


### THE DENTAL REGISTER.

VOL. LIII.] JUNE, 1899. [No. 6.

### COMMUNICATIONS.

### "The Cuspid Tooth in its Relation to Character."

BY DR. FRANK L. SCHATTUCK.

Read before the Detroit Dental Society.

*To the Dental Society*:—

To formulate a title to the subject of this paper prepared for your consideration, has been a somewhat difficult matter.

It has been my desire to make plain and comprehensive each leading factor in it, being fully sensible that the presentation here advanced will be somewhat startling to the mind on its first announcement.

The endeavor to explain the causes of the growth of the teeth will be looked upon as presumptuous; a large portion of mankind regarding such matters as beyond the scrutiny of human understanding and indeed scarcely falling within the field of legitimate inquiry.

It would be decidedly deleterious to the progress of truth to circumscribe the inquisitive and restless excursions of thought, with a broad line, marked prejudice, and confine ourselves to its baneful influence, would be an error indeed.

Man may err in his speculations, but it should be remembered that grievous errors frequently lie near important truths, and to the striving influence of the former leads to the happy discovery of the latter.

Therefore "The CuspidTooth in its Relation to Character" as the title, will, I hope, give us sufficient latitude, not only to speculate, but to use the modifications necessary to it.

The subject thus being defined the modification arising may be regarded as carrying its own lessons with it.

In the first consideration, then, it may be plainly derivable from such a theme as I have chosen ; that the teeth typically and beautifully illustrate the marvelous adaptation of animal forms to the varied ways and habits of life.

This wondrous fertility of contrivance which is characteristic of life at large is with all the many varities of animals, and their wild modes of living, the teeth are built to meet the exigencies they may require.

The cuspid tooth as a tooth of character is not such a hypothesis as at first we might be led to suppose.

You have only to remember its prominent position to at once acknowledge that facial characteristics at least with the human face depends very materially upon its presence.

Perhaps the term characteristic now should be meant as defining those exponents and proclivities, rather than the idiosyncrasy of an individual.

In making this discrimination I have in mind the characteristic differences between those formidable, cruel, snarling, and carnivorous beasts, contrasted with the herbivorous animals : the former having the cuspid largely developed in strength of purpose, while the latter have it developed as regards a sexual proclivity only : such as the wild boar, the babiroussa or Abyssinian hog, ferocious gorilla, wart-hog, and some others.

Writers, in attempting to account for these and analogous external structures, appear to satisfy themselves with the remark that they have been given for the purpose of self-defence, or to enable the animal to combat with greater advantage; but does this meet all the ends to which they are subservient?

Nature in this point of view is extremely partial in the distribution of her gifts.

It may be well for the eagle to possess talons; but we must not forget that these endowments are terrible in their effects upon a less favored portion of creation.

The elephant will enable us to place in a strong point of view the distinction between supplementary and essential organs ; and the facts which the investigation will elicit will tend to corroborate in a striking degree the correctness of the principles advocated.

It is obvious that the teeth are the seat of the predominant vital actions of the body. It is in them that life displays itself in the affluence of its energies.

We find in nature a great many animals who show modest and even timid disposition who have only the slightest rudiments of a cuspid tooth, or have it left out of their economy entirely.

Does this notable difference in the teeth mean anything as to strength, or indomitable selfishness or aggressive disposition?

Now, in regard to some peculiar developments of this tooth of character, we find in the males of many animals, the horse, "canines," or "bridle teeth," are present but are found wanting or only slightly developed (if found at all), in the mouth of the females.

In the hog the cuspids are relatively large (disproportionately) so in the male, in the upper jaw curve round in such a manner that the point of the crown is directed upward, truly a symbol of excessiveness. It is only by castration that abnormal growth of this tooth can be arrested, showing thereby that it is conditional to sexual influences.

The wart-hog has cuspids of enormous size; the superior ones are directed upward, piercing the upper lip and then curve backward toward the eye, their length is sometimes as much as eight or ten inches.

When the boar passes the age of sexual activity then it is these teeth with some others are shed.

In the African and other elephants we find the cuspid tooth absent.

The centrals or the incisors rather are greatly enlarged and elongated, the female of the African species has tusks quite as long as the male.

And too we find in studying the habits and natures of these colossal beasts, that they are found generally in pairs, showing true affection towards each other for life with little regard for sexual preferences.

To simply say that this is so, that this tooth with its several modifications from its simplest rudiment to its most excessive and complex development, without giving any reason why it is so, conveys little information.

It is not within the province of this paper to fully impart that information, but if one tooth is simple and another complex there is certainly a reason for it, and it is not only in the province of, but is clearly the duty of the odontologist to discover and point out these reasons if they can be found to exist.

With this brief comparative thought in regard to the animals, we will turn our thought into the field of human investigation and see what characteristic differences we find in not only the peculiar development but non-development as well.

The cuspid teeth, sometimes erronously called eye-teeth, are four in number situated at the angles of the mouth, and the superior cuspid on its labial surface is a little narrower in the mesio-distal direction than in the central incisor, with nearly equal length from the point of the cusp to the gingival line.

Instead of a straight or slightly curved cutting edge as in the incisors, the central portion of the crown is extended into a well-formed cusp, with the cutting edges sloping away to the mesial and distal angles.

Of these cutting edges the distal is a little longer than the mesial, and from the angle to the gingival line the distal surface is a little shorter than the mesial.

In unworn teeth, the angle formed by the union of the cutting edges to form the cusp is usually about ninety degrees or a square.

The lingual surface presents the same general marginal configurations as the labial; this surface is almost straight from the cusp to the linguo-gingival ridge, or singulum, but sometimes concave.

The marginal ridges arise from the mesial and distal angles and unite with the singulum. These ridges are usually large near the angles, and much less pronounced toward the gingival ridge.

The latter is prominent and is often raised into a tubercle, or cusp.

Occasionally this part of the enamel is thrown into irregular folds with grooves between, which are sometimes fissured. More rarely the small cusp may be divided by a groove; the linguo-gingival groove is often pronounced in unworn teeth.

The root of the upper cuspid is the longest in the human mouth; it is irregular conical in form.

In most examples the body of the root is straight and tapers to a slender point, which is often curved to the labial and distal, though the form of the root presents great variations. Frequently it is very crooked, perhaps, because, when it is taking its place in the arch, it is often crowded by the teeth mesial and distal to it, so that its growth in a right line is interrupted.

Thus is described a typical upper cuspid, of the several modifications; let us take some other examples aside from the typical.

All who have read Du Maurier's "Trilby" must have been impressed with the striking characteristic features of "Svengali."

"Svengali walking up and down the earth seeking whom he might cheat, betray, exploit, borrow money from, make brutal fun of, bully if he dared, cringe to if he must—man, woman, child or dog, was about as bad as they make 'em."

Du Maurier has only depicted in words what he has so nicely portrayed in the many illustrations of Svengali, found in his book—"Trilby."

Where we find the cuspids long and pointed with high festoon of the gums the gingival and middle thirds constituting the larger portion of the crown, so that the incisive third is very abrupt and pointed, the tooth perfectly straight on the mesial and distal approximal surface, then do we find a SVENGALI!

Take from the typical illustration or cartoons of our New York *Puck* and other papers of its class, and we notice how the artist portrays the features of the outlaw and banditti.

Notice in them the cruel expression caused by the curling of the lip over the cuspid tooth.

The cuspid developed so as to be easily disclosed by the levator muscles, as in haughtiness and scorn.

Whenever we find the motive temperament predominating in an individual, it not only shows earnestness of purpose, but determination, that falters never ; although often beset by sickness and other obstacles, which are overpowering in a great many other than this temperament where we observe a strong, round often curved cuspid.

True it is, physical growth can, and is changed by culture, which properly comes in another place other than this paper—we are speaking now of virgin nature, pure and simple.

Hastening now over these characters that declare themselves out of harmony with the laws of society, or the principles which govern the same, we will for further illustration regard the modification of these grosser organizations, who show ideality deficient, and compare those who have the creative disposition well developed—that is ideality.

Between these two classes as named we find the characters (as furnished by Chas' Dickens) viz. : "Uriah Heep," who are always "so very humble" that they never develop any cuspid teeth at all.

As the cuspids meet the highest ideal in arrangement that is in prosthetics, so in the natural arrangement it meets the requirements of a given case.

Just as youth is budding into the character of man, these teeth take their stand with the rest. And who is prepared to deny that their form, their location, whether regular or irregular, prominent or otherwise, are not the forerunner of the final development of the mind, as it establishes character in the body, over which is rules supreme.

Has it not been noticed when a deciduous cuspid remains past its time of erruption, it seems to take on a new lease of life, and crumpling up its succedaneous brother stands forth to do battle through life : What of it? Have you made note of the general character of such persons as possess them?

You will have noticed that they show a child-like disposition all their lives, and there is produced shape to the mouth that seems to sympathize with the same idea.

There is often seen a type of cuspid associated with a character described as "smooth," almost perfectly round in form, a little cusp extending down breaks the absolute circle.

The disposition of those persons who are characterized by this form of tooth can mold themselves into almost any condition of circumstance, or environment.

From a sly thief, society darling, or a type so adored by sympathetic woman who haven't much to do, the friend, the tame cat, the platonic lover (with many loves)—the squire of dames the trusty one, of whom husbands and brothers have no fear!—the delicate, harmless dilettante of Eros. The woman flatters and the man confides—and there is no danger whatever I am told—and I am glad!

As brevity is after all only a matter of suggestion, therefore in the biology of the cuspid tooth, I will only make brief suggestions, hoping that you will read between the lines.

Again there is a very long and decidedly pointed cuspid, characteristic of a discriminating mind, known to be peculiar, notional, strong in its likes and dislikes, are natural students of the laws of health, anatomy, chemistry, and physiology. Persons of great endurance and aptitude, in the acquisition of an education; their eyes are keen and discriminating, oftentimes taking in a whole page of reading matter at a glance, making very rapid proof-readers. They are great lovers of music, in fact, some musicians of high degree (observed by the writer) have this type of cuspid. Their judgement of color is excellent, and any discord or inharmony affects them very much, even destroying their appetite. They have a strong tendency to selfishness, and a disposition to control others is a characteristic running through their nature. Nansen, the Arctic explorer, is a representative of the above, and, I might add, has this type of cuspid.

Often we have a cuspid of complex form, of great length, extending from the gingival border to the point of the cusp, and of considerable width from mesial to distal angles.

It can be plainly discerned that a curved line, extended between the gingival third and the middle third, would make of it the round type or form.

Thus we have the sly thief—combined with the cunning, and artful disposition, or, in other words, our conception of a character known as the "American Red-man."

We all know full well of the Indian, his peculiarities, and it will not be required of me to enter into his history here. It has been the privilege of the writer to examine splendid specimens of this type of cuspid among these wild children of nature.

It would be an interesting story in itself, if I should relate to you the funny and queer experiences I have had while collecting evidences of corroborative data along these lines. The principles here put forth have been applied very frequently to individual men, woman and children; this gives me some confidence that others may find practical results from these studies, to not only aid us in a better understanding expression of the face, the features and meeting intelligently that demand made upon us as dentists; through loss of the teeth to select for, and arrange by the art of prosthesis, the restoration of the same.

I think the detailed and experimental method of studying expression may throw light, much light, upon the laws and processes of nutrition and evolution.

So it is here that we deal with the principles and modes of expression of hidden conditions as a matter preliminary to other studies.

Artists, and those who give literary descriptions of the mental and emotional conditions of man have taught us much, and we as dentists are alive to take every advantage thus offered and apply this knowledge with good results.

